# Supplementary material for: Academic performance of children in relation to gender, parenting styles, and socioeconomic status: What attributes are important
Source: PLoS One. 2023 Nov 15;18(11):e0286823. doi: 10.1371/journal.pone.0286823 (PMC10651006; doi:10.1371/journal.pone.0286823)
Supplement: Attachment — Submitted filename: Response to editor.docx [file pone.0286823.s001.docx]

Dear Dr. Ali,

We've checked your submission and before we can proceed, we need you to address the following issues:

1.Please ensure that you include a legends/captions for Figure 1 and 2 within your main document. We do appreciate that you have a legends in your uploaded figures however, we do require this to be part of the manuscript file itself.

Response-The figures is inserted in the revised manuscript
