# Supplementary material for: Academic performance of children in relation to gender, parenting styles, and socioeconomic status: What attributes are important
Source: PLoS One. 2023 Nov 15;18(11):e0286823. doi: 10.1371/journal.pone.0286823 (PMC10651006; doi:10.1371/journal.pone.0286823)
Supplement: Attachment — Submitted filename: Response to reviewers.docx [file pone.0286823.s002.docx]

**Revised comments**

1. I personally not satisfied with title; it does not look professional. This need to be revised and should be between 12-16 words.

Response: the title is revised accordingly to suggested range of words.

2. My suggestion is to avoid headings in abstract and this should be whole one paragraph, unless not required to journal.

Response: abstract is revised as suggested

3. Also revised the methodology section in the abstract because you have already revised the sampling techniques.

Response: methodology section in the abstract is revised as suggested and aligned with the sampling technique used.

4. The language in result section of abstract may be corrected, e.g. see the sentence. “The family socioeconomic status emerged as the strongest predictor of the academic performance of children (β=18.25) followed by the interaction of socioeconomic status and authoritative parenting (β=14.18), authoritative parenting (β=13.38), the interaction of socioeconomic status and permissive parenting (β=11.46), and permissive parenting (β=9.2).

Response: the paragraph iss revised while correcting the language.

5. The authors claim their response that introduction has been revised as per earlier comments, but I can’t see the comments incorporated. It is good that you reduced the introduction, but you did not introduce the aim and objectives of the study in introduction. The readers would be interested to quickly grasp the main purpose of research in the very beginning.

Response: aim and objectives of the study is included in the revised version

6. The section on “National and International Scenario “ may come before the theoretical debate and may be continued with introduction and this would then clarify my above comments on introduction.

Response: rearranged this section as suggested

7. The socioeconomic status and monthly income may be revised “(1=monthly income
less than PRs. 15, 000, 2=monthly income PRs. 15,000-30,000 and 3= monthly income above PRs.
30,000)“. Under international standard all the above categories fall below the poverty line and low socio-economic status. This may be inline with minimum wage labour PKRS, 25000, i.e. upto 25000, 26000 to 50000 and 51000 and above.

Response: the attributes of SES is aligned with minimum wage labour as suggested: PKRS, 25000, i.e. up to 25000, 26000 to 50000 and 51000 and above.

8. In conclusion, the second part of this sentences does not clarify the purpose that you are conveying “In addition, the family socioeconomic status emerged as the strongest predictor of the academic performance of children, followed by the interaction of socioeconomic status and authoritative parenting, the interaction of socioeconomic status and permissive parenting, authoritative parenting and permissive parenting” what you want to convey from this sentence??

Response: the sentence is explained and revised to make it clearer

9. Under recommendation you have added the following
“The findings also call for the mobilization of charitable resources like Zakat (religious binding on rich Muslims to distribute 2.5% of their financial resources like gold, silver and /or money among needy poor) and Ushr (religious binding on Muslim farmers to distribute 1/10th to 1/20th of their agriculture produce among needy poor) with a strong religious endorsement for supporting educational and other financial needs of families with low socioeconomic status” . How you relate this with your result and findings? Is there any such findings or analysis?? You may put recommendation only in relation to your variables.

Response: the recommendation was revised and related with the study findings on SES and academic performance.
