# Supplementary material for: Academic performance of children in relation to gender, parenting styles, and socioeconomic status: What attributes are important
Source: PLoS One. 2023 Nov 15;18(11):e0286823. doi: 10.1371/journal.pone.0286823 (PMC10651006; doi:10.1371/journal.pone.0286823)
Supplement: Attachment — Submitted filename: Response to reviewers.docx [file pone.0286823.s003.docx]

**Response to editor comments**

1. Rephrase the sentence in introduction section "*Also, when the state has more citizens with poor academic skills, they are not able to introduce productivity-enhancing technologies and new......*"

Response: rephrased the line in the revised version

2. Avoid the abbreviations such as etc

Response: the abbreviations are elaborated in the revised version

3. The sentence starting from "These factors include individual, social and institutional characteristics and so on" the phrase "so on" does not make any sense you have to explain all the factors or factors of your interest here in detail, the phrase "so on" makes the sentence ambiguous.

Response: corrected in the revised version

4. Aim and objectives should be presented in introduction section without heading and subheading(s). For this purpose a dedicated single paragraph may be used to present them.

Response: corrected as suggested

5. In methodology section, details on the study area must be added for the readers who are not familiar with local context and arrangements.

Response: more detail about the study area is provided in the revised version

6. Rephrase the sentence of Cronbach value of the scale used in this study that should indicate the value of each scale for this study within the accepted range.

Response: Cronbach value of each scale was added

7. Although it is well explained in the ethical statement that informed consent was taken from Head of the educational institute but little has been mentioned that how informed consent was taken from students.

Response: Cronbach value of each scale was added

8. A more detail may be added for the protocol of each interview.

Response: protocol of each interview was explained in the revised draft

9. Conflict of interest must be presented just after the conclusion.

Response: placed as suggested

10. The asterisk in Table 7 must be defined in parenthesis.

Response: the asterisk in Table 7 are defined in parenthesis

11. In discussion some sentences are quite interesting and confusing such as "Thus the democratic culture promotes............, The author added................., Researcher himself (which indicates that a single research involvement in this study). Check all these sentences for correction and relevance with your study.

Response: these sentences were revised and make it in-line with the study

12. In conclusion the authors claim that this study has policy implications but could not provide any detail on how and where aspects of policy implication.

Response: the policy implication is well highlighted in the revised draft

13. The author needs to argue that why gender variable is not significant in this study with scientific reason(s).

Response: the explanation for this is provided in the revised draft

14. Few practical recommendations may be present that should be within the mandate of legal and policy instruments. Irrelevant recommendation may be removed.

Response: Policy implications that were highlighted in the conclusions are also clubbed in the recommendations therefore, the recommendations is explained to make it aline with the conclusion and I think there is no irrelevant recommendation included

15. Please check reference number 2, 8, 13, 19, and 25.

Response: corrected
